# Placeboeffekte in der Schmerztherapie: Einfluss der Erwartung auf die Wirksamkeit und Verträglichkeit schmerzmedizinischer Behandlungen

**DOI:** 10.1007/s00482-022-00685-3

**Published:** 2023-01-13

**Authors:** Angelika Kunkel, Ulrike Bingel

**Affiliations:** Klinik für Neurologie, Zentrum für translationale Neuro- und Verhaltenswissenschaften, Universitätsklinikum Essen, Hufelandstr. 55, 45147 Essen, Deutschland

**Keywords:** Erwartungseffekte, Nocebo-Hyperalgesie, Behandlungserwartung, Schmerzwahrnehmung, Kommunikation, Expectation effects, Nocebo hyperalgesia, Treatment expectation, Pain perception, Communication

## Abstract

Erwartungen von Patienten beeinflussen die Wahrnehmung und neuronale Verarbeitung akuter und chronischer Schmerzen und modulieren die Wirksamkeit einer analgetischen Behandlung. Die Behandlungserwartung ist nicht nur die wichtigste Determinante der Placeboanalgesie. Behandlungserwartungen beeinflussen auch Wirksamkeit und Verträglichkeit „aktiver“ pharmakologischer und nichtpharmakologischer Schmerzbehandlungen. Jüngste Erkenntnisse über die psychologischen und neurobiologischen Mechanismen, die den Effekten von Behandlungserwartungen zugrunde liegen, ermöglichen und fordern die systematische Integration und Modulation von Behandlungserwartungen in schmerzmedizinischen Behandlungskonzepten. Eine solche Strategie verspricht, die Schmerztherapie zu optimieren und die Belastung durch unerwünschte Nebenwirkungen sowie den Missbrauch von Analgetika zu verhindern oder zu verringern. Diese Übersicht beleuchtet aktuelle Konzepte, neueste Errungenschaften, aber auch Herausforderungen und offene Forschungsfragen.

## Abstract (en)

Expectations of patients influence the perception and neuronal processing of acute and chronic pain and modulate the effectiveness of analgesic treatment. The expectation of treatment is not only the most important determinant of placebo analgesia. Expectations of treatment also influence the efficacy and tolerability of “active” pharmacological and non-pharmacological treatment of pain. Recent insights into the psychological and neurobiological mechanisms underlying the clinically relevant effects of treatment expectations enable and call for the systematic integration and modulation of treatment expectations into analgesic treatment concepts. Such a strategy promises to optimize analgesic treatment and to prevent or reduce the burden of unwanted side effects and the misuse of analgesics, particularly of opioids. This review highlights the current concepts, recent achievements and also challenges and key open research questions.

## Lernziele

Nach Lektüre dieses Beitrags

- kennen Sie die psychologischen und neurobiologischen Einflussfaktoren von Placeboeffekten in der Schmerztherapie.
- wissen Sie um die Bedeutung von Placebo- bzw. Erwartungseffekten im Rahmen von aktiven beispielsweise pharmakologischen oder anderen spezifischen schmerztherapeutischen Behandlungen.
- sind Ihnen situationsabhängige und personenbezogene Einflussfaktoren der Erwartungshaltung sowie deren Einfluss auf den Schmerz und analgetische Therapien bekannt.
- können Sie besser einordnen, warum manche Behandlungsansätze nicht zum erwarteten Behandlungserfolg führen.
- können Sie Aspekte der Arzt-Patienten-Kommunikation und Aufklärung einsetzen, um die Behandlungserwartungen des Patienten zugunsten des Behandlungsergebnisses zu beeinflussen.

## Hintergrund

Studien der letzten Jahrzehnte zeigen, dass die Erwartungen von Patienten in Bezug auf die Wirksamkeit einer Behandlung einen entscheidenden Einfluss auf die Gesundheit und den Erfolg medizinischer Behandlungen haben (umfassende Übersichtsartikel siehe [1, 2, 3, 4, 5, 6]). Eine zentrale Rolle der Erwartung lässt sich am besten anhand von Studien mit (inaktiven) **Placebobehandlungen**

Placebobehandlungen

veranschaulichen. Neben dem natürlichen Verlauf einer Erkrankung kann eine Verbesserung der Symptomatik hier nicht durch spezifische Eigenschaften eines Arzneimittels erklärt werden, sondern die Erwartung der Patienten an die Behandlung ist entscheidend. Metaanalysen randomisierter, placebokontrollierter klinischer Studien („randomised controlled trials“ [RCT]) haben bei unterschiedlichen Erkrankungen gezeigt, dass ein Großteil der **Symptomverbesserung**

Symptomverbesserung

auf Placeboeffekte zurückzuführen ist [7, 8]. Diese werden in verschiedenen **physiologischen Systemen**

Physiologische Systeme

und bei verschiedenen Erkrankungen beobachtet [6], ihre **Effektstärken**

Effektstärken

scheinen von System zu System zu variieren. Sehr große Effekte findet man bei der Behandlung von Schmerzen und Depressionen, bei denen bis zu 70 % des gesamten Therapieerfolgs auf Placeboeffekte zurückgeführt werden können [9, 10].

Erwartungen können auch **negative Auswirkungen**

Negative Auswirkungen

auf das Behandlungsergebnis haben, was als **Noceboeffekt**

Noceboeffekt

bezeichnet wird. Es ist davon auszugehen, dass ein Großteil der von Patienten in klinischen Studien gemeldeten unerwünschten Symptome gar nicht durch das Arzneimittel selbst verursacht ist, sondern im Zusammenhang mit **negativen Erwartungen**

Negative Erwartungen

oder **Vorerfahrungen**

Vorerfahrungen

steht. Darauf deuten systematische Reanalysen der Placeboarme in klinischen Studien hin, die belegen, dass unerwünschte Wirkungen nicht nur in der Häufigkeit des Auftretens, sondern auch in der Art der Symptomatik der aktiven Behandlung ähneln [10, 11, 12]. Nocebostudien zeigen, dass die bloße Erwartung intensiverer oder häufigerer Schmerzen die **Schmerzempfindlichkeit**

Schmerzempfindlichkeit

auf neuronaler und Verhaltensebene modulieren kann [13, 14, 15, 16, 17, 18]. Negative Erwartungen spielen ebenfalls eine wichtige Rolle bei der Entwicklung und Aufrechterhaltung neuer Symptome, wie in einer Studie der Arbeitsgruppe um Benedetti zum Höhenkopfschmerz deutlich wurde. Die Studienteilnehmer, die von einem anderen Teilnehmer zuvor darüber informiert worden waren, dass der Aufenthalt in der Forschungsstation auf 3500 Höhenmetern zu **Höhenkopfschmerzen**

Höhenkopfschmerzen

führen könne, zeigten eine signifikante Zunahme der Prävalenz und Intensität von Kopfschmerzen. Interessanterweise zeigten diese Versuchsteilnehmer auch veränderte **Prostaglandinspiegel**

Prostaglandinspiegel

im Blut, ein physiologisches Korrelat des über psychosoziale Faktoren induzierten Kopfschmerzes in großer Höhe [19]. In ähnlicher Weise kann die bloße Erwartung, dass die Schmerzempfindlichkeit im Laufe der Zeit zunehme, der natürlich auftretenden **Habituation**

Habituation

an wiederholte schmerzhafte Reize bei gesunden Versuchsteilnehmern entgegenwirken und zu einer **Schmerzsensibilisierung**

Schmerzsensibilisierung

führen [20].

## Einfluss der Erwartung auf aktive (analgetische) Behandlungen

Die **individuelle Erwartung**

Individuelle Erwartung

beeinflusst die Wirksamkeit und Verträglichkeit aktiver medizinischer, beispielsweise **pharmakologischer Behandlungen**

Pharmakologische Behandlungen.

**Figure 1:**
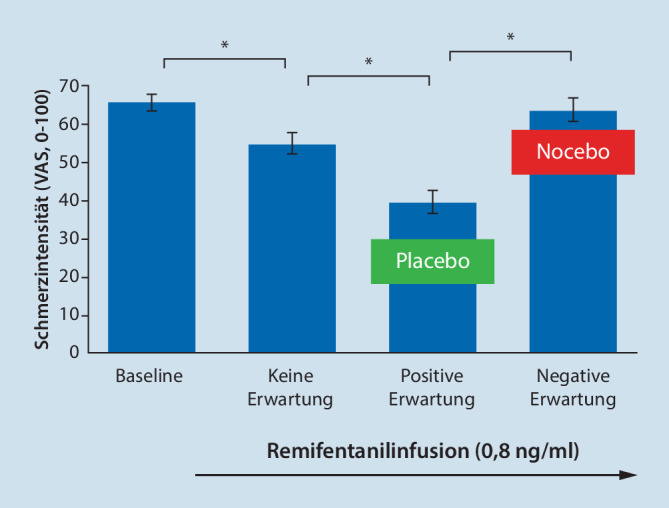

Eine positive Behandlungserwartung kann die schmerzlindernde Wirkung eines potenten Opioids ([14]; Abb. 1), die Wirksamkeit eines Triptans in der Akutbehandlung der Migräne [21] wie auch den Erfolg einer medikamentösen Migräneprophylaxe steigern [22]. Bis zu 50 % dieser Effekte analgetischer Behandlungen können auf die Erwartung und nicht auf die pharmakologische Wirkung des verabreichten Medikaments zurückgeführt werden [14, 22, 23, 24, 25]. Dies gilt auch für **nichtmedikamentöse Interventionen**

Nichtmedikamentöse Interventionen,

wie den Erfolg einer interdisziplinären multimodalen Schmerztherapie [26, 27].

Im Gegensatz dazu kann eine negative Erwartungshaltung die therapeutische Wirkung pharmakologischer Behandlungen verringern [14, 28] und wesentlich zur Entwicklung **unerwünschter Nebenwirkungen**

Unerwünschte Nebenwirkungen

beitragen [29, 30]. Der Vergleich der Nebenwirkungsprofile in klinischen Studien hat gezeigt, dass sowohl die **Qualität**

Qualität

als auch die **Quantität**

Quantität

der berichteten Nebenwirkungen in den Placebogruppen denen der aktiven Behandlungsgruppen ähneln [11, 31, 32, 33]. Darüber hinaus ist die Umstellung von **Originalpräparaten**

Originalpräparate

auf **Generika**

Generika

mit identischen Wirkstoffen häufig mit einer Zunahme unerwünschter Ereignisse verbunden und kann so einen Abbruch der Behandlung begünstigen [34], ein Effekt, der kürzlich auch für die Umstellung von Referenzbiologika auf Biosimilars diskutiert wurde [35].

### Merke

Erwartungen können nicht nur die Reaktion auf inerte Behandlungen (Placebos), sondern auch die Wirksamkeit und Verträglichkeit aktiver medizinischer Behandlungen, einschließlich der Pharmakotherapie, erheblich beeinflussen. Hierbei kann es nicht nur zu einer Verbesserung (Placeboeffekt), sondern auch zu unerwünschten Auswirkungen der Behandlungserwartung auf das Behandlungsergebnis kommen (Noceboeffekt). Der Grad des Einflusses variiert zwischen Individuen und physiologischen Systemen.

## Psychologische Determinanten der Behandlungserwartung

**Figure 2:**
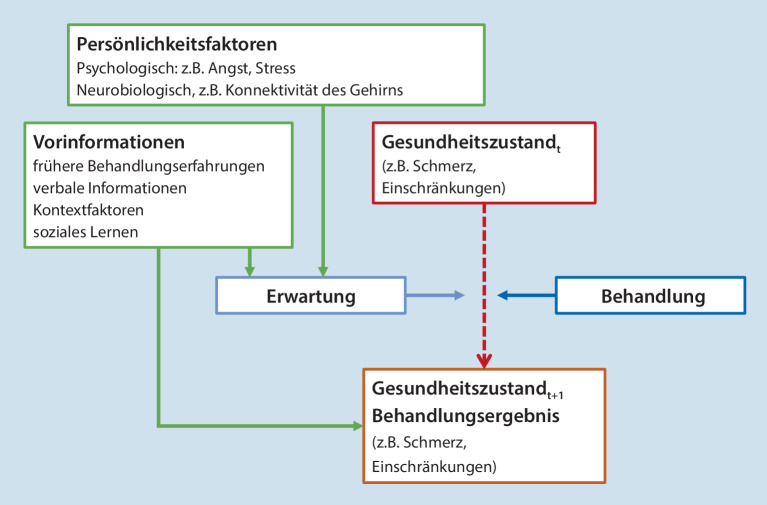

Die Behandlungserwartung der Patienten wird durch folgende Faktoren hervorgerufen oder beeinflusst (Abb. 2):

- Verbale Informationen
- Frühere Behandlungserfahrungen
- Das Beobachten von Therapieerfolg und -misserfolg bei anderen
- Merkmale des therapeutischen Kontexts oder der therapeutischen Maßnahme selbst

Verbale Informationen über die erwarteten Wirkungen von Behandlungen werden sowohl durch die direkte **mündliche Kommunikation**

Mündliche Kommunikation

mit Angehörigen der Gesundheitsberufe als auch durch **schriftliche Informationen**

Schriftliche Informationen

im Rahmen von Einwilligungserklärungen oder durch den Beipackzettel für Medikamente erteilt [14, 21, 33, 36]. Digitale und soziale Medien spielen dabei eine zunehmend wichtige Rolle [37, 38]. Mithilfe **assoziativer Lernparadigmen**

Assoziative Lernparadigmen

wurde gezeigt, dass Vorerfahrungen mit analgetischen Behandlungen die Behandlungserwartung induzieren oder verstärken, wobei implizite und explizite Lernprozesse in unterschiedlichem Ausmaß beteiligt sein können [39, 40]. Neuere Erkenntnisse deuten darauf hin, dass sich diese Effekte im Laufe der Zeit und über verschiedene Arten von Behandlungen hinweg generalisieren können [41, 42].

Die wiederholte Exposition gegenüber pharmakologischen Wirkstoffen kann eine Reaktion hervorrufen, die die Wirkungen des Medikaments selbst nachahmt, auch wenn schließlich nur ein Placebo verabreicht wird. Dieses Phänomen wird auch als **pharmakologische Konditionierung**

Pharmakologische Konditionierung

bezeichnet [44, 45, 46, 47] und kann sowohl die erwünschten Wirkungen eines Medikaments als auch unerwünschte Wirkungen umfassen. Experimentelle Studien deuten darauf hin, dass die Beobachtung positiver oder negativer Behandlungseffekte auf Schmerzen bei anderen die Behandlungserwartungen und -ergebnisse beeinflussen kann, das heißt, dass Behandlungserfahrungen nicht unbedingt aus erster Hand stammen müssen [48, 49, 50, 51, 52]. Zusätzlich werden sie auch durch die Merkmale des **therapeutischen Kontexts**

Therapeutischer Kontext

und des **medizinischen Umfelds**

Medizinisches Umfeld

beeinflusst, etwa durch das Auftreten des Behandelnden oder die klinische Umgebung [53]. Darüber hinaus spielen auch die Merkmale der Behandlung selbst eine Rolle [54]. So wecken beispielsweise **invasive Eingriffe**

Invasive Eingriffe,

wie Akupunktur oder Chirurgie, stärkere Behandlungserwartungen und in der Folge größere Placeboeffekte als weniger invasive Behandlungen, wie orale Therapien mit Tabletten [55]. In ähnlicher Weise können **aktive Placebos**

Aktive Placebos

– das heißt Behandlungen, bei denen eine pharmakologische Substanz verabreicht wird und wahrnehmbare (Neben‑)Wirkungen hervorruft, aber keinen Einfluss auf das Zielsymptom hat – stärkere Placeboeffekte hervorrufen als **inerte Placebos**

Inerte Placebos

[56]. Diese Ergebnisse regen zu weiteren Untersuchungen an, um zu erforschen, wie Merkmale der Behandlung und des Behandlungskontexts zugeschnitten werden können, um die positiven Behandlungserwartungen zu optimieren [57].

### Merke

Behandlungserwartungen können auf vielfältige Weise hervorgerufen und beeinflusst werden, insbesondere durch verbale Informationen, frühere Behandlungserfahrungen, das Beobachten von Therapieerfolg und -misserfolg bei anderen, aber auch durch Merkmale des therapeutischen Kontexts oder der therapeutischen Maßnahme selbst.

## Neurobiologische Mechanismen von Erwartungseffekten auf Schmerz

In den vergangenen drei Dekaden wurden die neurochemischen und neurobiologischen Mechanismen der Effekte von positiver und negativer Erwartung auf die Schmerzwahrnehmung in sogenannten Placebo- und Noceboparadigmen umfangreich untersucht. Hierbei beschäftigte sich der überwiegende Teil der Studien mit der **Placeboanalgesie**

Placeboanalgesie

und den Effekten der positiven Erwartung. Die **Nocebohyperalgesie**

Nocebohyperalgesie

ist bislang deutlich schlechter untersucht. Das kann zumindest teilweise durch die ethischen und klinischen Einschränkungen erklärt werden, die mit der absichtlichen Induktion negativer Erwartungen, insbesondere bei Patienten, verbunden sind.

**Neuroimaging-Studien**

Neuroimaging-Studien

zeigen, dass die Placeboanalgesie absteigende, schmerzmodulierende Bahnen involviert, darunter der dorsolaterale präfrontale Kortex (**DLPFC**

DLPFC),

der rostrale anteriore zinguläre Kortex (**rACC**

rACC)

und das periaquäduktale Grau (**PAG**

**Figure 3:**
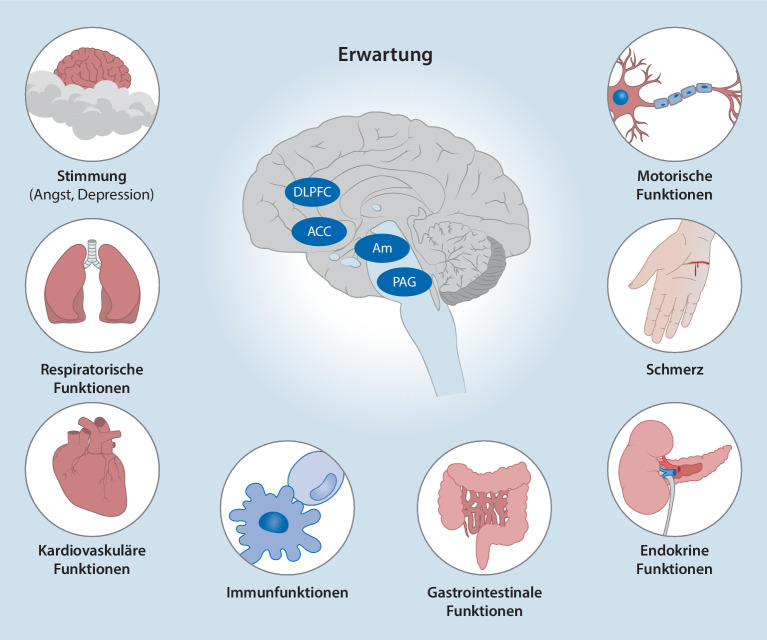

PAG;

[58, 59, 60]; Abb. 3). Diese Areale scheinen über **Top-down-Prozesse**

Top-down-Prozesse

die Aktivität in **schmerzverarbeitenden Hirnarealen**

Schmerzverarbeitende Hirnareale,

wie der Insula und dem somatosensorischen Kortex, zu modulieren. Mehrere Studien dokumentieren zudem, dass Placeboanalgesie und Nocebohyperalgesie mit einer veränderten Verarbeitung nozizeptiver Reize im Rückenmark einhergehen [18, 61]. Eine vorübergehende Läsion des DLPFC kann die Placeboanalgesie reduzieren [62]. Darüber hinaus sind die Degeneration und verminderte Konnektivität der Frontallappen bei der Alzheimer-Krankheit mit einem beeinträchtigenden oder vollständigen Verlust der erwartungsinduzierten Analgesie verbunden [63]. Zusammengenommen deuten diese Befunde darauf hin, dass der DLPFC maßgeblich an der Generierung und Aufrechterhaltung der Wirkung von Behandlungserwartungen auf Schmerzen beteiligt ist. Neuere Erkenntnisse lassen außerdem vermuten, dass die anteriore Insula die Erwartung und die nozizeptive Verarbeitung integriert [64, 65].

Obwohl die oben genannten Studien einen Zusammenhang zwischen Placeboanalgesie und einer reduzierten nozizeptiven Verarbeitung hergestellt haben, unterstreicht eine kürzlich durchgeführte Metaanalyse von Studien mit **funktioneller Magnetresonanztomographie**

Funktionelle Magnetresonanztomographie

(fMRT), dass dieser Mechanismus nicht ausreicht, um die analgetischen Effekte auf Verhaltenseffekte zu erklären [66]. Denn tatsächlich zeigte sich eine erhebliche Diskrepanz zwischen den Placeboeffekten auf subjektiver Ebene und den Effekten der Placebobehandlung auf die sogenannte **neurologische Schmerzsignatur**

Neurologische Schmerzsignatur

[67], ein validiertes Maß für die nozizeptive Signalverarbeitung im Gehirn. Dies deutet darauf hin, dass ein großer Teil der Placeboeffekte über Hirnmechanismen vermittelt wird, die unabhängig von einer Modulation der afferenten nozizeptiven Verarbeitung sind und eher die kognitive und emotionale Bewertung von Schmerzen betreffen.

Was die beteiligten **Neurotransmitter**

Neurotransmitter

betrifft, so scheinen **endogene Opioide**

Endogene Opioide

von zentraler Bedeutung für die Placeboanalgesie zu sein [25, 68, 69, 70]. Außerdem wurde auch das mesolimbische dopaminerge System mit der Placeboanalgesie in Verbindung gebracht, weil es bei Placeboanalgesie zu einer erhöhten **dopaminergen Neurotransmission**

Dopaminerge Neurotransmission

kommt, wobei dessen spezifische Rolle bisher noch nicht geklärt ist [71]. Ferner korreliert die individuelle Placeboanalgesie mit dem **Belohnungsverhalten**

Belohnungsverhalten

[17] und mit den strukturellen Eigenschaften des Striatums [72]. Allerdings führt die pharmakologische Blockade von Dopamin nicht zu einer Schwächung der Placeboanalgesie [73, 74], was darauf hindeutet, dass die Rolle des dopaminergen mesolimbischen Systems bei der Placeboanalgesie komplexer ist. Während die oben genannten Studien gegen eine direkte analgetische Wirkung sprechen, ist das dopaminerge System vermutlich für andere der Placeboanalgesie inhärente Prozesse wie das Belohnungslernen von Bedeutung.

Erste Studien zu negativen Erwartungseffekten zeigen, dass Erwartungen, welche Schmerzen verstärken, mit einer erhöhten Aktivität in Arealen der **somatosensorischen Schmerzverarbeitung**

Somatosensorische Schmerzverarbeitung,

einschließlich des Rückenmarks, einhergehen [15, 18]. Pharmakologische Studien haben die Nocebohyperalgesie mit einer Aktivierung des **endogenen Cholecystokininsystems**

Endogenes Cholecystokininsystem

in Verbindung gebracht, eines Systems, das neurobiologisch eng mit dem Konstrukt der Angst verbunden ist, die wiederum ein starker Modulator der Nocebohyperalgesie ist [75]. Bildgebende Studien haben eine erhöhte Aktivität und funktionelle Konnektivität des **Hippocampus**

Hippocampus

und der **Amygdala**

Amygdala

gezeigt [14, 76], was ebenfalls die Annahme der Beteiligung angstbezogener Netzwerke bei Nocebohyperalgesie unterstützt.

Insgesamt sind die neurobiologischen Mechanismen zwar zunehmend, aber lange nicht vollumfänglich verstanden. Insbesondere Unterschiede zwischen den Mechanismen positiver und negativer Erwartungseffekte, aber auch zwischen gesunden Versuchsteilnehmern und Patienten mit chronischen Schmerz- oder anderen, beispielsweise neurodegenerativen, Erkrankungen sind bislang nur unzureichend verstanden. Darüber hinaus ist nicht bekannt, ob die bei akuten experimentellen Schmerzen während einer einzigen Sitzung beschriebenen Mechanismen auch für die Erwartungseffekte über längere Zeiträume hinweg verantwortlich sind, wie etwa bei Patienten mit chronischen Schmerzen.

### Merke

An der Placeboanalgesie sind sowohl intrakortikale als auch deszendierende Netzwerke beteiligt. Dazu gehören insbesondere der DLPFC, der rACC und das PAG. Auf neurochemischer Ebene sind insbesondere endogene Opioide und das dopaminerge mesolimbische System beteiligt.

## Die Herausforderung: interindividuelle Unterschiede bei den Auswirkungen der Erwartung auf Schmerz und Analgesie

Die Auswirkungen von Erwartungen auf den Behandlungserfolg variieren beträchtlich zwischen Individuen. Bis heute sind wir nur begrenzt in der Lage, die Behandlungserwartungen einer Person in einer bestimmten klinischen Situation, die dynamischen Veränderungen dieser Erwartungen über die Zeit und die Effekte der Erwartung auf Therapien vorherzusagen. Erkenntnisse darüber, wie stark ein Patient von einer positiven Erwartungshaltung profitiert, hätten unmittelbare Auswirkungen auf die klinische Versorgung. So könnte bei Patienten, die unter positiven Erwartungen die endogene Schmerzmodulation ausreichend aktivieren können, eine **geringere Analgetikadosis**

Geringere Analgetikadosis

erforderlich sein, während andere **höhere Dosen**

Höhere Dosen

benötigen würden, wie bereits bei Patienten mit Alzheimer-Krankheit gezeigt wurde [63]. Die Kenntnisse über **Persönlichkeitsfaktoren**

Persönlichkeitsfaktoren

(sogenannte State- und Trait-Faktoren), die die Effekte der Behandlungserwartung auf den Schmerz modulieren oder sogar vorhersagen, sind daher von entscheidender Bedeutung, wenn therapeutische Entscheidungen optimal und personalisiert angepasst werden sollen. Studien haben gezeigt, dass **Angst**

Angst,

**Stress**

Stress

und **negativer Affekt**

Negativer Affekt

sowohl die Entwicklung als auch die Effekte positiver Erwartung modulieren können [77, 78, 79]. Gleichzeitig kann Angst die schmerzverstärkende Wirkung negativer Behandlungserwartungen [80] und die Wirkung positiver Erwartungen auf die Opioidanalgesie verringern [14]. Auch lang anhaltende **Angststörungen**

Angststörungen

und **Depressionen**

Depressionen

scheinen eine Rolle zu spielen [16, 81]. Bislang wurden interindividuelle Unterschiede allerdings nur in Studien mit vergleichsweise kleinen Stichprobengrößen untersucht, was die widersprüchlichen Ergebnisse erklären könnte [4].

Auch bei **neurobiologischen Faktoren**

Neurobiologische Faktoren,

wie der individuellen Gehirnstruktur und -funktion, konnte gezeigt werden, dass die Integrität der weißen Substanz innerhalb des DLPFC sowie zwischen dem DLPFC und Schlüsselbereichen des schmerzmodulierenden Netzwerks, beispielsweise rACC und PAG, interindividuelle Unterschiede in der Placeboanalgesie erklären kann [82]. Ebenso haben mehrere fMRT-Studien gezeigt, dass bei chronischen Schmerzzuständen die funktionelle Konnektivität des präfrontalen Kortex und anderer Hirnregionen im Ruhezustand die Reaktion auf psychologische und pharmakologische Behandlungen vorhersagen kann [83, 84, 85, 86, 87, 88]. **Genetische Faktoren**

Genetische Faktoren

wie Variationen in relevanten Neurotransmittersystemen (siehe oben) tragen dazu bei, wie ein Individuum auf Behandlungserwartungen bei Schmerzen reagiert [89, 90].

### Merke

Aktuell gibt es keine verlässlichen Prädiktoren, welche die individuelle Ausprägung einer Erwartung oder deren Effekt auf Schmerz und Analgesie vorhersagen können. Zweifelsohne spielen aber psychologische, neurobiologische und genetische Faktoren eine Rolle.

## Neue Entwicklungen: Open-label-Placebobehandlungen

Das **ethische Dilemma**

Ethisches Dilemma

in der „traditionellen“ Anwendung von Placebobehandlungen liegt darin, dass der Patient über das Wesen der Placebobehandlung im Unklaren gelassen wird. Ein solches Vorgehen ist selbstverständlich ethisch und juristisch unzulässig und mit den Prinzipien der **Patientenautonomie**

Patientenautonomie

wie auch der **vertrauensvollen Kommunikation**

Vertrauensvolle Kommunikation

zwischen Arzt und Patient nicht zu vereinbaren. Aktuelle Strategien der sogenannten **offenen Placebobehandlung**

Offene Placebobehandlung

bzw. Open-label-Placebo(OLP)-Behandlung umgehen dieses Dilemma, indem der Patient vor einer Behandlung mit Placebos über das Wesen der Placebobehandlung informiert wird.

Erste klinische **Proof-of-concept-Studien**

Proof-of-concept-Studien

zeigen, dass auch eine offene Darreichung von Placebos wirksam sein kann. Tatsächlich belegt mittlerweile eine Vielzahl von Studien positive Effekte solcher OLP-Behandlungen auf experimentelle, akute und verschiedene chronische **Schmerzerkrankungen**

Schmerzerkrankungen

[91], aber auch auf andere, insbesondere **subjektive Beschwerden**

**Figure 4:**
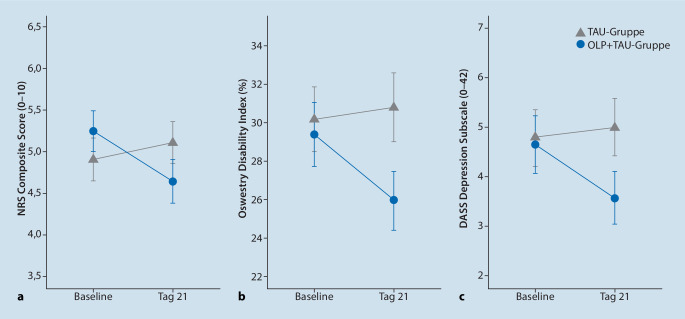

Subjektive Beschwerden

wie Fatigue oder depressive Symptome. Positive Effekte einer 3‑wöchigen OLP-Behandlung auf die Schmerzintensität, die Beeinträchtigung sowie den Schmerzmittelgebrauch bei chronischen unspezifischen Rückenschmerzen (Abb. 4; [92, 93]) wurden bereits in unabhängigen Untersuchungen demonstriert. Die Mechanismen, die der Wirkung von OLP-Behandlungen zugrunde liegen, Langzeiteffekte sowie Prädiktoren für ein Ansprechen auf diese Behandlung sind Gegenstand aktueller wissenschaftlicher Untersuchungen.

## Unerforschte Territorien: Wie kombinieren sich oder interagieren die Effekte von Erwartung und aktiver (pharmakologischer) Behandlung?

**Figure 5:**
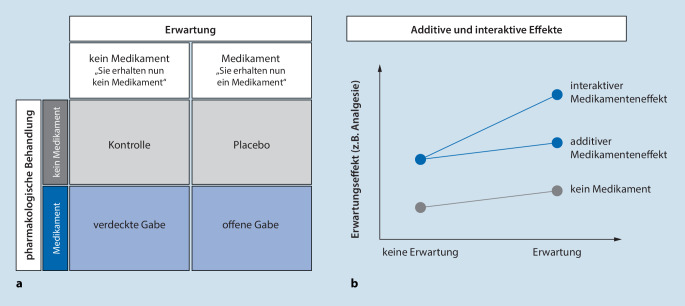

Eine wichtige, noch unbeantwortete Frage ist, ob die Wirkungen von Erwartung und Behandlung additiv oder interaktiv zusammenwirken (Abb. 5b). Pharmakologische Mechanismen und die Erwartung, die endogene neurobiologische Kaskaden auslöst, könnten substanz- bzw. behandlungsabhängig additiv, aber auch interaktiv zusammenwirken. Die sogenannten „balanced placebo designs“ ([94]; Abb. 5a), die sowohl ein Placebo als auch eine aktive Behandlung umfassen, bieten die einzigartige Möglichkeit, die Mechanismen und Auswirkungen der Behandlungserwartung, der Behandlung selbst und ihrer Interaktion zu beschreiben. Bisherige Studien mit diesem Design deuten auf die Existenz interaktiver bzw. multiplikativer Effekte hin, die zu **synergistischen Effekten**

Synergistische Effekte,

aber auch **subadditiven Effekten**

Subadditive Effekte

von Behandlungserwartungen und pharmakologischer Analgesie führen können [95]. Auch wenn die Frage, ob sich die Effekte von Erwartung/Placebo und Verum additiv oder interaktiv verhalten, zunächst von rein akademischer Natur zu sein scheint, so haben diese Erkenntnisse fundamentale Implikationen für die Konzeption und Interpretation placebokontrollierter klinischer Studien. Denn diese beruhen auf der (möglicherweise falschen) Grundannahme, dass sich die Effekte von Placebo und Verum ausschließlich additiv verhalten (siehe auch [4, 6]).

## Systematische Nutzung von Behandlungserwartungen im klinischen Umfeld

Auch wenn noch viele offene Forschungsfragen bestehen, sind die vorliegenden Belege für einen substanziellen Beitrag von Erwartungseffekten zu Behandlungsergebnissen überzeugend genug, dass Erwartungen an die Behandlung selbst Ziel **systematischer Interventionen**

Systematische Interventionen

im klinischen Kontext sein müssen, um die Behandlungsergebnisse zu verbessern. Hierbei sind die Behandlungserwartungen der Patienten als **dynamische Konstrukte**

Dynamische Konstrukte

zu verstehen, die auf der Grundlage von Vorinformationen entstehen, wobei unterschiedliche Ebenen des impliziten und expliziten Lernens und des Bewusstseins beteiligt sind [5, 17]. Mit anderen Worten: Behandlungserwartungen sind formbar und können systematisch verändert werden, indem die verfügbaren Informationen über die Behandlung moduliert werden (praktische Empfehlungen siehe Tab. 1). Hierbei spielt die **Arzt-Patienten-Kommunikation**

Arzt-Patienten-Kommunikation

eine zentrale Rolle.

**Table 1:** 

Einen authentischen und einfühlsamen Kommunikationsstil pflegen
Die Ängste, Sorgen und Behandlungserwartungen der Patienten regelmäßig bewerten und ansprechen
Angemessene Informationen über Krankheiten, Diagnosen und Behandlungen bereitstellen
Stellen Sie proaktive Rückfragen (bitten Sie die Patienten, die bereitgestellten Informationen zusammenzufassen), um negative Vorurteile und Missverständnisse zu vermeiden
Bereitstellung einer „offenen Medikation“
Verstärkung positiver Assoziationen und Minimierung negativer Assoziationen zwischen der therapeutischen Intervention und kontextuellen Faktoren
Optimierung der Behandlungserwartung und der Erwartung unerwünschter Wirkungen, aber Vermeidung von Erwartungsverletzungen
Ausgewogene Darstellung von erwünschten Behandlungseffekten, unerwünschten Wirkungen und Rahmeninformationen über Nebenwirkungen zur Minimierung von Noceboeffekten
Vermittlung und Training von Strategien zur Bewältigung negativer Auswirkungen
Verweisen Sie auf webbasierte und andere Informationssysteme, die evidenzbasierte Informationen anstelle von unbewiesenen, angstschürenden Kommentaren liefern
Gestaltung, Layout und Inhalt der Packungsbeilagen von Arzneimitteln verbessern, einschließlich der Mechanismen und gezielten Wirkungen von Arzneimitteln, der Laiensprache und der patientenorientierten Darstellung von Wahrscheinlichkeiten (grafisch statt numerisch)
Nutzen Sie das Lernen durch Beobachtung, z. B. durch Peer-to-peer-Coaching oder Videoclips mit Patienten, die gut auf eine schmerzlindernde Behandlung ansprechen
Bieten Sie multisensorische Behandlungshinweise (z. B. visuell, olfaktorisch, gustatorisch) in Verbindung mit dem aktiven Medikament, um Konditionierungsprozesse zu fördern

### Verbale Informationen

Verbale Informationen zu Symptomen, Erkrankungen und Therapien sind in der klinischen Versorgung allgegenwärtig. Wie im Paradigma der offenen und verdeckten Medikation dargestellt (Abb. 5a), kann allein das Wissen, dass eine Behandlung stattfindet und welche gezielten Medikamente eingenommen werden, den Behandlungserfolg substanziell modulieren. Das heißt, ein wichtiger erster Schritt wäre hier sicherzustellen, dass jeder Patient zu jedem Zeitpunkt über das Wesen seiner Erkrankung und Behandlung und darüber, wie und wann eingenommene Medikamente wirken, informiert ist. Die positiven Effekte einer guten Kommunikation und der Bereitstellung angemessener Informationen wurden nicht nur in experimentellen Studien, sondern auch in ersten klinischen Proof-of-concept-Studien belegt. Dies konnte für schmerztherapeutische Kontexte wie die Schmerzbehandlung nach Brust- [96] oder Knieoperation [97], aber auch im Zusammenhang mit schweren somatischen Grunderkrankungen, wie bei der Genesung nach einem Myokardinfarkt [98] oder nach elektiven Herzoperationen [99], gezeigt werden. Eine authentische und empathische Kommunikation, positive Formulierungen (**Framing**

Framing)

und eine gezielte, **patientenzentrierte Aufklärung**

Patientenzentrierte Aufklärung

sind einfach anzuwendende Strategien, um die Behandlungserwartung und deren Effekt auf den Therapieerfolg zu optimieren. Diese Maßnahmen können also als wichtiges Merkmal der Schmerzbehandlung angesehen werden, wie bereits in den nationalen Behandlungsleitlinien hervorgehoben wird [100].

### Frühere Behandlungserfahrungen

Frühere Behandlungserfahrungen haben einen starken Einfluss auf die gegenwärtigen Behandlungserwartungen. Während im **experimentellen Setting**

Experimentelles Setting

die Wirksamkeit einer Behandlung erfolgreich moduliert und damit das Ansprechen auf diese verstärkt werden kann, ist das im **klinischen Umfeld**

Klinisches Umfeld

meist schwierig. Oft steht keine wirksame Therapie zur Verfügung, um eine positive Behandlungserfahrung zu induzieren, insbesondere bei chronischen Schmerzzuständen. Ärzte und medizinisches Fachpersonal sollten sich deshalb der nachteiligen Auswirkungen früherer negativer Erfahrungen bewusst sein, die sich über die Zeit und die Vielzahl an Behandlungsansätzen verallgemeinern können [41, 101]. Die Berücksichtigung früherer Erfahrungen und Präferenzen der Patienten in Bezug auf eine Analgetikabehandlung sollte deshalb in das Behandlungskonzept integriert werden. Die systematische Modulation von Behandlungserfahrungen (Abb. 5), beispielsweise durch eine pharmakologische Konditionierung, konnte bei akutem Schmerz nach wiederholter Verabreichung von Nichtopioid- und Opioidanalgetika gezeigt werden [102, 103], stellt jedoch bei chronischen Schmerzzuständen aktuell noch eine Herausforderung dar. Nichtpharmakologische Ansätze, beispielsweise Entspannungstechniken, können ebenfalls systematisch mit pharmakologischen Analgetika gekoppelt werden, um die erlernte Analgesie bei nachfolgenden Behandlungen zu nutzen. Aktuelle Ansätze explorieren sogar, wie Lernmechanismen in Kombination mit offenen Placebos genutzt werden können, um die Wirksamkeit von Methadon bei behandlungsbedürftigen Opioidabhängigen zu erhöhen [104, 105].

### Beobachtung von Behandlungseffekten

Behandlungserwartungen müssen nicht zwingend durch eigene Erfahrungen erworben werden, sondern können auch durch die Beobachtung von Behandlungseffekten bei anderen erzielt werden [48, 50]. Untersuchungen bei Patienten mit chronischen Rückenschmerzen weisen darauf hin, dass dieser Mechanismus auch die Wirksamkeit von Koanalgetika erhöhen kann [52]. Das **Beobachtungslernen**

Beobachtungslernen

könnte daher eine besonders nützliche Strategie für Patienten sein, für die keine wirksame Behandlung verfügbar ist. In künftigen experimentellen und klinischen Studien sollte deshalb untersucht werden, ob und wie die Beobachtung des Behandlungserfolgs eines anderen Patienten dazu genutzt werden kann, positive Erwartungen an eine Behandlung und die anschließenden Erfolge in klinischen Populationen zu verbessern. Hier könnten digitale Ansätze vielversprechend sein, beispielsweise durch das Beobachten standardisierter Videos von exemplarischen positiven Behandlungsverläufen.

An dieser Stelle muss darauf hingewiesen werden, dass die absichtliche Induktion einer zu optimistischen bzw. unrealistisch positiven Erwartung durch die Behandelnden weder hilfreich noch ethisch vertretbar ist. Vielmehr könnten sich Verletzungen dieser übersteigerten Erwartung durch ausbleibenden Behandlungserfolg (sogenannter „prediction error“) nicht nur negativ auf das **Vertrauensverhältnis**

Vertrauensverhältnis

zwischen Behandler und Patient auswirken, sondern auch den Behandlungseffekt selbst negativ beeinflussen. Daher ist es von großer Bedeutung, dass sich therapeutische Modifikationen der Behandlungserwartungen an **vorbestehenden Erwartungen**

Vorbestehende Erwartungen

des Einzelnen und an den individuell **realistischen Behandlungsergebnissen**

Realistische Behandlungsergebnisse

orientieren.

## Fazit für die Praxis

- Behandlungserwartungen sind nicht nur die treibende Kraft von Placebo- und Noceboeffekten. Sie können akute und chronische Schmerzen bedeutsam beeinflussen und die Wirksamkeit und Verträglichkeit von analgetischen Behandlungen modulieren. Sie gehen mit messbaren Veränderungen der Schmerzwahrnehmung und -modulation im zentralen Nervensystem einher. Sowohl das Opioid- und Dopaminsystem als auch individuelle psychologische Determinanten spielen dabei eine wichtige Rolle.
- Durch eine gute, verständliche und patientenzentrierte Aufklärung und Bereitstellung von Information, eine empathische Zuwendung, positive Formulierungen und einen professionellen therapeutischen Rahmen können Sie als Behandler bereits jetzt die positive Erwartungsbildung und damit auch den Behandlungserfolg positiv beeinflussen.
- Die in diesem Beitrag dargestellten neurobiologischen und psychologischen (Lern‑)Prozesse sollen Ihnen ein tieferes Verständnis der objektivierbaren Mechanismen und Auswirkungen von Behandlungserwartungen auf den Behandlungserfolg sowie deren therapeutische Relevanz vermitteln. Sie sollen dazu ermutigen, die Behandlungserwartung von Patienten als festen Bestandteil in die Behandlung zu integrieren. So können Sie dazu beitragen, individuelle Behandlungseffekte zu verbessern sowie die Belastung durch unerwünschte Nebenwirkungen und den Missbrauch von Analgetika zu verringern.

## CME-Fragebogen

Was versteht man unter Placeboeffekten?

Den natürlichen Verlauf einer zugrunde liegenden Erkrankung mit den dazugehörigen Schwankungen

Folgemessungen von Patienten mit initial extrem hohen (oder niedrigen) Werten, die dann häufig im Normbereich liegen

Positive physiologische oder psychologische Effekte, die auf die Erwartungshaltung, assoziative Lernprozesse und eine gute Arzt-Patienten-Kommunikation zurückzuführen sind

Scheinantworten, die keinen statistisch relevanten Effekt auf ein bestehendes Modell haben

Statistisches Rauschen in klinischen Studien durch unkontrollierbare Störvariablen

Ein 82-jähriger Patient mit multiplen internistischen Erkrankungen stellt sich mit einer Postzosterneuralgie bei Ihnen vor. Welche der genannten Überlegungen ist richtig?

Altersabhängige Effekte in der Pharmakokinetik und -dynamik können bei der Wahl der schmerztherapeutischen Strategie vernachlässigt werden.

Mögliche Wechselwirkungen mit den internistischen Medikamenten des Patienten müssen bei der Wahl der schmerztherapeutischen Strategie nicht beachtet werden.

Sie bemühen sich um den Einsatz von Kommunikationsstrategien, um die Wirksamkeit und Verträglichkeit der von Ihnen verordneten Therapie zu optimieren.

Sie behandeln den Patienten mit einem Vitaminpräparat, das Sie als „Placebo“ einsetzen, ohne ihn über das Wesen der Placebobehandlung zu informieren.

Im Hinblick auf unerwünschte Wirkungen von topisch wirksamen Medikamenten ziehen Sie vorrangig systemisch wirksame Ansätze in Betracht.

Welche Aussage in Bezug auf die psychologischen Mechanismen bei Placeboeffekten trifft zu?

Verbal induzierte Erwartung und Vorerfahrung/Konditionierung wirken unterschiedlich stark auf verschiedene körperliche Systeme.

Frühere Behandlungserfahrungen von Patienten haben nur wenig Einfluss auf die Behandlungserwartungen.

Der Einfluss von Erwartungs- und Lernmechanismen ist in allen körperlichen Systemen gleich.

Placeboeffekte früherer Behandlungserfahrungen werden nicht auf andere Behandlungsansätze generalisiert.

Behandlungserwartungen werden erst durch eigene Erfahrungen mit einer bestimmten Behandlung erzeugt.

Welcher Mechanisums/Vorgang liegt der Placeboanalgesie zugrunde?

Die Placeboanalgesie beruht auf psychologischen Mechanismen.

Sie führt zu einer Veränderung der peripheren Schmerzweiterleitung.

An der Placeboanalgesie sind vorwiegend aszendierende Schmerzbahnen beteiligt.

Wichtige Prozesse der Placeboanalgesie finden vorwiegend im Kleinhirn statt.

Sie ist mit der Aktivierung körpereigener schmerzhemmender Systeme assoziiert.

Was versteht man unter einem „balanced placebo design“?

Es untersucht die Effekte der optischen Aufmachung eines Generikums.

Es untersucht die Wirkung psychotroper Substanzen in verschiedenen Applikationsformen.

Es erlaubt die Untersuchung von Erwartungseffekten auf die Wirksamkeit von aktiven pharmakologischen Substanzen.

Es gehört zu den Untersuchungen vor Markteinführung eines neuen Medikaments.

Es demonstriert, dass die Erwartung für die Wirksamkeit von Medikamenten keine Rolle spielt.

Ein 89-jähriger Patient mit zentralen neuropathischen Schmerzen nach Schlaganfall hat schon viele frustrane Behandlungsversuche hinter sich. Welcher Aspekt ist besonders wichtig für Ihr Gespräch mit dem Patienten?

Sie betonen, dass die Vorbehandler vermutlich nicht so gut ausgebildet waren wie Sie, und loben Ihre langjährige Expertise als Schmerztherapeut.

Sie erklären dem Patienten, dass diese Art von Schmerzen sehr schwierig zu behandeln ist.

Sie behandeln mit einem Placebo, da Medikamente ja nicht geholfen haben.

Sie sind sich der Bedeutung der Vorerfahrungen, Ängste und Sorgen des Patienten bewusst und adressieren den Einfluss dieser Faktoren im Gespräch.

Im Hinblick auf die vorherigen negativen Erfahrungen verzichten Sie komplett auf die Erläuterung möglicher unerwünschter Wirkungen.

Was sollten Sie über unerwünschte Wirkungen eines Medikaments wissen?

Eine detaillierte Aufklärung der Patienten über alle möglichen Nebenwirkungen verhindert unerwünschte Wirkungen.

Metaanalysen zeigen, dass ein Großteil der Nebenwirkungen von Medikamenten auch in den Placeboarmen auftritt.

Frühere Behandlungserfahrungen mit dem gleichen Medikament haben keinen Einfluss auf unerwünschte Wirkungen.

Unerwünschte Wirkungen bei der Umstellung auf ein Generikum sind immer eingebildet.

Das Auftreten von unerwünschten Wirkungen ist ausschließlich durch das pharmakologische Profil von Medikamenten bestimmt.

Was gilt es bei der Arzt-Patienten-Kommunikation zu berücksichtigen?

Sie hat nur einen sehr geringen Einfluss auf den therapeutischen Erfolg einer Behandlung.

Sie ist überbewertet und sollte aus dem Lernzielkatalog von Medizinstudierenden entfernt werden.

Sie ist ein wichtiger Einflussfaktor von Placeboeffekten und sollte gezielt eingesetzt werden, um die Wirksamkeit und Verträglichkeit von medizinischen Behandlungen zu unterstützen.

Sie sollte für alle Patienten möglichst standardisiert erfolgen.

Sie sollte ihren Fokus auf die Nebenwirkungen einer Behandlung, die in der Packungsbeilage beschrieben werden, richten.

Welches Verständnis über Placeboeffekte ist essenziell für die klinische Nutzung?

Placeboeffekte treten nicht nur im Rahmen von wissenschaftlichen Placebobehandlungen auf, sondern auch im Rahmen pharmakologischer oder anderer spezifischer Behandlungen, die den Erfolg einer Therapie substanziell beeinflussen können.

Ein Mittel, das die gleichen Symptome wie eine Erkrankung hervorrufen kann, kann in stark verdünnter Dosis keine positiven Effekte auf den Verlauf dieser Erkrankung haben.

Das Ausmaß von Placeboeffekten lässt sich nach heutigem Kenntnisstand sehr gut vorhersagen.

Placeboeffekte lassen sich selbst unter kontrollierten Bedingungen nicht von der pharmakodynamischen Wirkung eines Arzneimittels isolieren.

Placeboeffekte sind unbeeinflusst von verbalen Informationen des medizinischen Personals oder durch Vorerfahrungen mit anderen Behandlungen.

Was weiß man über die neurobiologischen Grundlagen der Placeboanalgesie?

Körpereigene Opioide sind im Zusammenhang mit der Placeboanalgesie kaum untersucht.

Der rostrale anteriore zinguläre Kortex (rACC), der dorsolaterale präfrontale Kortex (DLPFC) und das periaquäduktale Grau (PAG) sind wichtige Hirnareale bei der Entstehung der Placeboanalgesie.

Die dopaminerge Neurotransmission ist während der Nocebohyperalgesie signifikant erhöht.

Bei der Placeboanalgesie handelt es sich um Bottom-up-Prozesse.

Durch das Blocken des dopaminergen Systems kommt es zu einer vollständigen Aufhebung der Placeboanalgesie.
